# Ethanolic Extracts from *Azadirachta indica* Leaves Modulate Transcriptional Levels of Hormone Receptor Variant in Breast Cancer Cell Lines

**DOI:** 10.3390/ijms19071879

**Published:** 2018-06-26

**Authors:** Deisi L. Braga, Sara T. S. Mota, Mariana A. P. Zóia, Paula M. A. P. Lima, Priscila C. Orsolin, Lara Vecchi, Júlio C. Nepomuceno, Cristina R. Fürstenau, Yara C. P. Maia, Luiz Ricardo Goulart, Thaise G. Araújo

**Affiliations:** 1 Laboratory of Genetics and Biotechnology, Institute of Biotechnology, Federal University of Uberlandia, Uberlandia-MG 38700-128, Brazil; braga.deisi@gmail.com (D.L.B.); saratsm.s@hotmail.com (S.T.S.M.); 2 Laboratory of Nanobiotechnology, Institute of Biotechnology, Federal University of Uberlandia, Uberlandia-MG 38400-902, Brazil; marianazoia@hotmail.com (M.A.P.Z.); laravecchi7@yahoo.it (L.V.); yaracpmaia@gmail.com (Y.C.P.M.); lrgoulart@ufu.br (L.R.G.); 3 Laboratory of Cytogenetic and Mutagenesis, University Center of Patos de Minas, Patos de Minas-MG 38700-207, Brazil; paulamarynella@hotmail.com (P.M.A.P.L.); priscilaco@unipam.edu.br (P.C.O.); nepomuceno@ufu.br (J.C.N.); 4 Laboratory of Animal Cell Culture, Institute of Biotechnology, Federal University of Uberlandia, Uberlandia-MG 38700-128, Brazil; cfurstenau@ufu.br; 5 University of California Davis, Dept. of Medical Microbiology and Immunology, Davis, CA 95616, USA

**Keywords:** breast cancer, *Azadirachta indica*, neem, ethanolic extracts, androgen receptor variant-7

## Abstract

Breast Cancer (BC) encompasses numerous entities with different biological and behavioral characteristics, favored by tumor molecular complexity. *Azadirachta indica* (neem) presents phenolic compounds, indicating its potential as an antineoplastic compound. The present study aimed to evaluate the cellular response of MCF10, MCF7, and MDA-MB-231 breast cell lines to ethanolic extracts of neem leaves (EENL) obtained by dichloromethane (DCM) and ethyl acetate (EA) solvent. Extracts’ antiproliferative activities were evaluated against MCF 10A, MCF7, and MDA-MB-231 for 24 and 48 h using MTT assay. *ESR1*, *ESR2*, *AR*, *AR-V1*, *AR-V4,* and *AR-V7* transcripts were quantified through qPCR for 0.03125 μg/mL of DCM and 1.0 μg/mL for EA for 48 h. The EENL was tested on *Drosophila melanogaster* as a sole treatment and then also together with doxorubicin. Antiproliferative effect on tumor cell lines without affecting MCF 10A were 1.0 µg/mL (*P* < 0.001) for EA, and 0.03125 µg/mL (*P* < 0.0001) for DCM, both after 48 h. Transcriptional levels of *AR-V7* increased after treatment. In vivo assays demonstrated that EENL induced fewer tumors at a higher concentration with doxorubicin (DXR). The behavior of *AR-V7* in the MDA-MB-231 tumor lineage indicates new pathways involved in tumor biology and this may have therapeutic value for cancer.

## 1. Introduction

According to the World Health Organization (WHO) [1], Breast Cancer (BC) is the most common malignant tumor among women. An increase from 14 million in 2012 to 22 million new cases is estimated by 2022. By 2035, the number of deaths is expected to grow by 70% [2]. BC is a disease that encompasses numerous entities, with peculiar biological and behavioral characteristics favored by a complex molecular microenvironment [3].

Malignancy results from regulatory imbalances involving pathways closely related to growth and proliferation [4,5,6,7]. In this context, receptors for estrogen and androgenic hormones are important proteins in cancer progression and play key roles in deciding the appropriate treatments [8].

Estrogen hormone binds to receptors on the nucleus membrane and regulates the expression of genes associated with survival, proliferation, and differentiation of mammary cells [9]. The estrogen receptor alpha (ESR1) is the main receptor responsible for these events, thus hormone therapy is considered when tumors display this receptor. However, therapies using the ESR1 as a target may cause some patients to become more resistant [8,10]. The ESR1 protein forms a homodimer or a heterodimer with the protein ESR2 (estrogen receptor beta) and is responsible for regulating gene function [11,12,13].

The *AR* gene encodes for the androgen receptor, which is a transcription factor activated by a steroid hormone [14,15]. Structurally related to ESR1, this protein is expressed in 80% of breast tumors, of which 55% are ESR1-positive and 35% are classified as triple-negative tumors (negative for ESR, progesterone receptor, and HER2 receptor). In recent years, 18 *AR* variants (*AR-V1* to *AR-V18*) have been described and characterized, especially focusing on their role in disease progression [16,17]. In prostate cancer, *AR-V7* is involved in cancer cell growth in the absence of androgens, which represents a highly advanced form of the disease [14,15,18,19]. From a clinical perspective, *AR* can be a favorable prognostic indicator [20], but its role in BC needs a deeper understanding [21,22,23,24].

In light of the regulatory role of ESR and AR, agents able to modulate these receptors’ gene expression emerges as a fundamental strategy for tumor aggressiveness control, and could potentially be used as new therapies.

Brazil has approximately 25% of the world’s biodiversity, providing great opportunities for the development of cancer drugs and therapies [25]. Different natural products present antitumor properties, endorsing the importance of scientific studies that elucidate their mode of action [26,27,28,29].

Among these diverse plants, the extracts from the species *Azadirachta indica* A. Juss., commonly known as “neem”, have been used for the treatment of inflammation, viral infections, hypertension, and displays insecticidal, nematicide, and fungicidal properties [30,31,32,33]. Although the bioactive compounds present in neem are found in different tissues of this plant, those from their seeds and leaves are more concentrated, accessible, and easily obtained by water or organic solvents extraction methods, such as those that use hydrocarbons, alcohols, ketones, or ethers [34,35].

Considering that natural phytochemicals contain phenolic compounds with antimetastatic activity [36,37,38,39], *A. indica* should be investigated in cancer research, since phenolic compounds were found in this species [40]. Balasenthil et al. (1999) [41] demonstrated that neem leaves extract administered to hamsters with oral carcinoma promoted tumor suppression by modulating lipid peroxidation, antioxidant action, and detoxification. Leaves of this species are also capable of activating an immune response [42]. It has also been reported that flavones isolated from neem flowers have antimutagenic effects by inhibition of the enzymatic activation of heterocyclic amines [43].

In this study, we hypothesized that ethanolic extracts from *Azadirachta indica* leaves (EENL) obtained by dichloromethane (DCM) or ethyl acetate (EA) extraction could modulate the expression of estrogen and androgen receptors, thus promoting molecular changes that would hinder the mammary tumor activity. Therefore, our goal was to evaluate the cytotoxic and mutagenic effects of the extracts and their effect on the expression of genes coding for the hormonal receptors in the lineages MCF 10A (non-tumorigenic), MCF7 (ESR + BC), and MDA-MB-231 (triple-negative BC [TNBC]).

## 2. Results

### 2.1. Bioactive Compounds and Antiproliferative Effects of EENL

Total phenols of DCM and EA extracts were calculated according to the standard curve of gallic acid equivalents (GAE) subjected to a linear regression. Concentrations of this bioactive compound were 40.415 ± 0.566 mg GAE/g and 45.200 ± 0.569 mg GAE/g for EA and DCM, respectively (*P* < 0.01). Antiproliferative activity of EENL extracts was further investigated in three breast lineages (MCF 10A, MCF7, and MDA-MB-231) through MTT assay.

**Figure 1:**
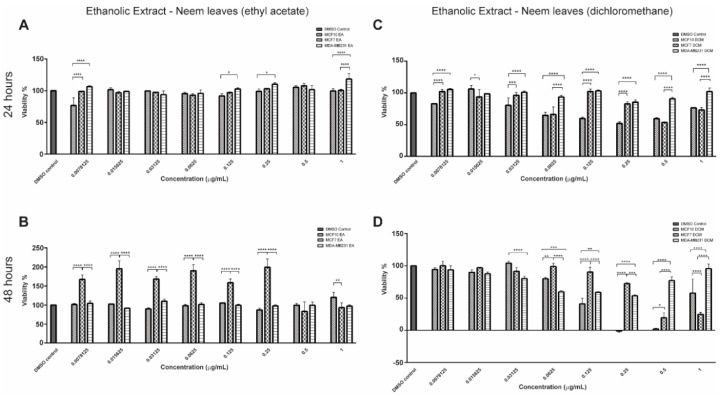
Effect of Ethanolic Extract of Neem Leaves (EENL) prepared from *A. indica* on breast cells (MCF 10A, MCF7, and MDA-MB-231) proliferation. (**A**,**B**) Treatment with EENL obtained with Ethyl Acetate (EENL–EA) extract for 24 and 48 h, respectively. (**C**,**D**) Treatment with EENL extract obtained using dichloromethane (EENL–DCM) for 24 and 48 h respectively. * *P* < 0.05, ** *P* < 0.01, *** *P* < 0.001 and **** *P* < 0.0001.

EENL–EA did not reduce the proliferation of breast cancer cell lines (Figure 1A) after 24 h of treatment. The non-tumorigenic lineage was more sensitive to the EA extract at 0.0078125 μg/mL, 0.125 μg/mL, 0.25 μg/mL, and 1.0 μg/mL. In addition, the viability of MCF7 increased after treatment at 0.0078125 μg/mL up until 0.25 μg/mL for 48 h (Figure 1B). However, in the highest concentration, MCF7 viability decreased compared to MCF 10A (*P* < 0.001).

The treatment with EENL–DCM extract for 24 h reduced the proliferation of MCF7 compared to MCF 10A (*P* < 0.05) at 0.015625 μg/mL (Figure 1C). After 48 h (Figure 1D), DCM extract inhibited the proliferation of the triple-negative tumor cell line (MDA-MB-231), with extracts’ concentrations ranging from 0.0625 to 0.25 μg/mL, when compared to MCF7 lineage. Compared to MCF 10A, the ESR + tumor cell line (MCF7) showed a reduction in viability at a concentration of 1.0 μg/mL. Only at the concentration 0.03125 μg/mL and 0.0625 were there significant differences (*P* < 0.001) between the non-tumorigenic lineage (MCF 10A) and the triple-negative cells’ (MDA-MB-231) viability.

**Figure 2:**
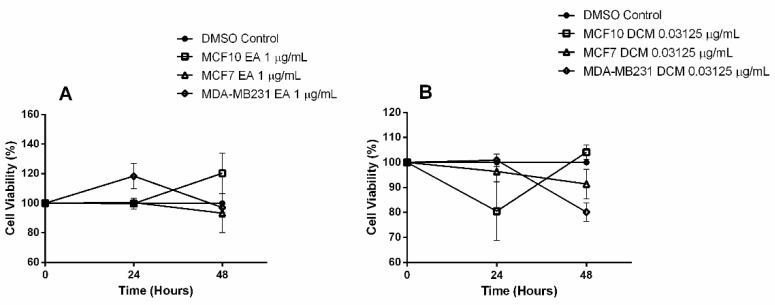
Time-course response to Ethanolic Extract of Neem Leaves (EENL). Treatment with 1.0 μg/mL of EENL obtained with ethyl acetate (EENL–EA) (**A**), and EENL obtained with dichloromethane (EENL–DCM) (**B**).

Based on these results, the concentrations that showed antiproliferative effect on tumor cell lines, but did not decrease MCF 10A viability, were chosen for molecular assays. For EENL–EA extract we used 1.0 µg/mL (*P* < 0.001), and for EENL–DCM extract 0.03125 µg/mL (*P* < 0.0001) after 48 h of treatment, as demonstrated in the time-course Figure 2.

### 2.2. Transcriptional Profile of Hormone Receptors after Treatment with EENL

**Figure 3:**
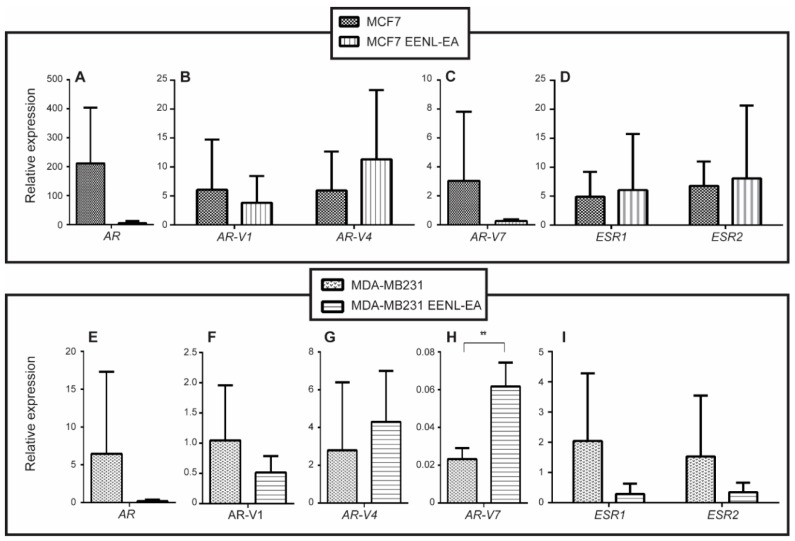
Relative expression of hormone receptors transcripts after treatment with Ethanolic Extract of Neem Leaves (EENL). Cell lines were treated with 1.0 µg/mL of EENL obtained with ethyl acetate (EENL–EA) for 48 h. Relative gene expression levels were evaluated for *AR* (**A**), *AR-V1*, *AR-V4* (**B**), *AR-V7* (**C**), *ESR1* and *ESR2* (**D**) in MCF7 cell line. In MDA-MB-231 the gene expression levels were evaluated for *AR* (**E**), *AR-V1* (**F**), *AR-V4* (**G**), *AR-V7* (**H**), *ESR1* and *ESR2* (**I**). The analyses were performed using the comparative Cq calibrated with data from MCF 10A and ** *p* < 0.01.

Gene expression of *ESR1*, *ESR2*, *AR*, *AR-V1*, *AR-V4,* and *AR-V7* in breast cells was evaluated before and after 48 h of treatment with 1.0 μg/mL of EENL–EA extract (Figure 3). *AR-V7* expression increased 2.85-fold (*P* < 0.01) in treated MDA-MB-231 cells after 48 h (Figure 3H). The remaining genes in MDA-MB-231, and all genes in MCF7, did not display statistically significant differences in gene expression levels. Although not significant, the mean *AR*, *AR-V1*, *ESR1,* and *ESR2* relative gene expression levels were higher in 48 h-treated MDA-MB-231 cells (31.74, 2.03, 7.07, and 4.37-fold, respectively).

**Figure 4:**
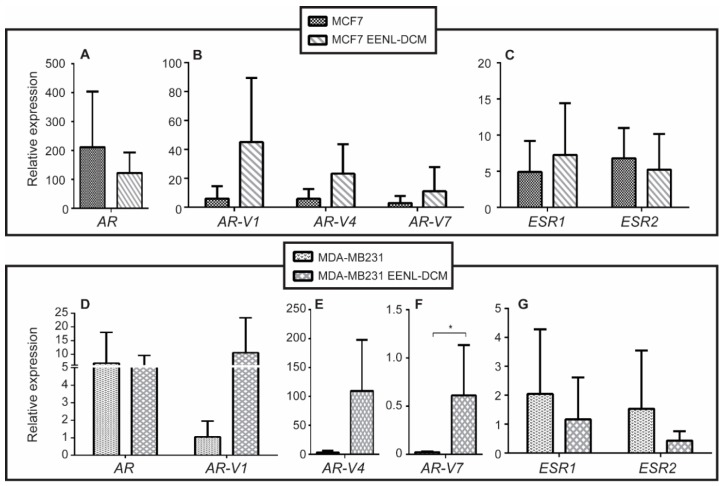
Relative expression of hormone receptors genes after treatment with Ethanolic Extract of Neem Leaves (EENL). Cell lines were treated with 0.03125 µg/mL of EENL obtained using dichloromethane (EENL–DCM) for 48 h. Relative gene expression levels were evaluated for *AR* (**A**), *AR-V1*, *AR-V4*, and *AR-V7* (**B**), *ESR1* and *ESR2* (**C**) in MCF7 cell line. In MDA-MB-231 the relative gene expression levels were evaluated for *AR* and *AR-V1* (**D**), *AR-V4* (**E**), *AR-V7* (**F**), *ESR1* and *ESR2* (**G**). The analyses were performed using the comparative Cq calibrated with data from MCF10 * *P* < 0.05.

No significant gene expression changes were found in MCF7 cells treated with EENL–DCM extract at 0.03125 μg/mL for 48 h (Figure 4). However, the TNBC cell line MDA-MB-231 had a 28.41-fold increase on the expression of *AR-V7* upon treatment (*P* < 0.05) (Figure 4F).

### 2.3. In Vivo Experiments with Drosophila Melanogaster

**Figure 5:**
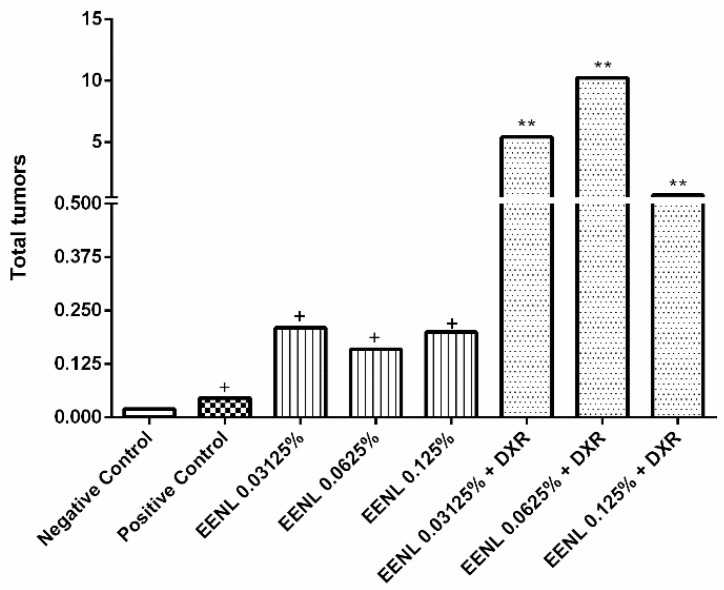
Frequency of tumors in *Drosophila melanogaster*. *Drosophila melanogaster* heterozygous for the warts tumor suppressor gene were treated with different concentrations of isolated Ethanolic Extract from Neem Leaves (EENL) (0.03125%, 0.0625%, and 0.125%) and associated with doxorubicin (DXR) (0.03125%, 0.625%, and 0.125%). The frequency of tumors was analyzed in different segments (eyes, head, wings, body, legs, and halteres). Statistical analysis was according to Mann–Whitney *U*-test with a significance level of *P* < 0.05. + value considered statistically different from the negative control (*P* < 0.05) and ** value considered statistically different from the positive control (DXR 0.4 mM) (*P* < 0.05).

The results showed the carcinogenic action of EENL isolate at the concentrations 0.03125%, 0.0625%, and 0.125%, and the modulating effect of the EENL on the carcinogenic action of doxorubicin (DXR at 0.4 mM). DXR may intercalate on DNA and induce formation of DNA adducts at active promoter sites, increasing torsional stress and enhancing nucleosome turnover. Furthermore, it may trap topoisomerase II at breakage sites, causing double strand breaks. Enhanced nucleosome turnover might increase the exposure of DNA to reactive oxygen species (ROS) resulting in DNA damage and cell death [44]. The frequency of the tumor clone per segment of *Drosophila melanogaster* is demonstrated in Table 1 and Figure 5.

The study design included a negative control, which was flies with mutated gene; and DXR as a positive control. For the negative control, the frequency of 0.02 of tumors per fly were observed, and this discrete tumor induction occurs due to the genetic predisposition of the test organism. On the other hand, the positive control induced a frequency of 0.46 of tumors per fly, proving that the organism lineage responded to tumor induction.

Larvae that were exposed to the EENL isolate, at concentrations of 0.03125%, 0.0625%, and 0.125% displayed frequencies of 0.21, 0.16, and 0.20 tumors per fly, respectively. Compared to the negative control, a statistically significant increase in tumor induction confirmed the carcinogenic effect of the extract on *D. melanogaster*. When 0.03125%, 0.0625%, and 0.125% of EENL were applied together with doxorubicin at 0.4 mM, the frequencies of tumors per fly were 5.43, 10.24, and 0.69, respectively. These results demonstrate that EENL with DXR increased tumor frequency at the lowest concentrations, when compared to the positive control. However, in the highest concentration used (0.125%), even when associated with DXR 0.4 mM, the tumor frequency decreased.

## 3. Discussion

The association between phenolic compounds in plants and antioxidant activity is due to phenolic hydroxyl groups that have a strong free radical scavenger activity [45,46,47,48,49,50]. Abdelhady et al. (2015) [40], during a phytochemical search for active substances, demonstrated that several phenolic compounds are present in *Azadirachta indica.*

In fact, natural phytochemicals contain phenolic compounds with the ability to prevent cancer metastasis [36]. Several studies have shown that different flavonoids and polyphenols exert an anti-metastatic effect [37,38,39]. Here we evaluated the cellular effects of ethanolic extracts of neem in breast cell lines. The presence of phenolic compounds in the EENL–DCM and EENL–EA demonstrate their potential antiproliferative activity, which may reduce tumor aggressiveness. The different cellular responses, evaluated by the MTT reduction, after treatments with EENL extracts obtained using DCM and EA, indicated this behavior. For the assays with *Drosophila melanogaster*, the EENL extracts induced higher tumors at a higher concentration when applied with DXR, probably due to the toxic effect of EENL together with the chemotherapeutic DXR, inducing cell death, which makes the appearance of tumors unfeasible.

According to Paul et al. (2011) [51], the major secondary metabolites present in the *Azadirachta indica* leaves are nimbolide, vilasinin, nimbinene, 6-deacetyl nimbinene, nimbandiol, nimocinol, β-sitosterol, β-sitosterol-β-d-glicoside, neem leaf glycoprotein, quercetin, glycoside of quercetin, glycoside of kaemferol, quercetin-3-galactoside (hyperin), and rutin. Nimbolide is the most abundant tetranortriterpenoid isolated from leaves of *A. indica*, showing apoptotic and antiproliferative activity acting in the following pathways: (1) oxidative stress and caspase activation; (2) reduction of the expression of anti-apoptotic proteins (Bcl-xl; Bcl-2) and increasing the expression of pro-apoptotic proteins (Bax, Bad, Bid, cytochrome c); (3) activation of the tumor suppressor p53; (4) activation of the extrinsic apoptosis pathway; (5) inhibition of IGF-1; (6) reducing levels of cyclin-dependent kinase (CDKs) and cyclins, promoting cell cycle arrest; and (7) inhibition of NFκB and its pro-tumorigenesis pathway [52,53,54]. The cytotoxicity profile verified in the MTT assays may also be attributed to the probable induction of apoptosis by the EENL. MCF7 cells were more sensitive to high concentrations of EENL. This behavior was previously observed in prostate cancer, in which hormone-responsive cells (LNCaP) were more sensitive to treatment with this extract [55]. Therefore, analysis of the gene expression, especially of hormone receptor genes, may indicate molecular patterns involved in EENL modulation in breast tumor cells.

The results of Aleskandarany et al. (2016) [56] have shown that there is an association between the expression of *AR* and good prognosis in BC. Comparing *AR* expression in HER+, TNBC, and luminal tumors, they observed that luminal BC presented a higher receptor expression. However, AR is an oncogene in triple-negative tumors by “replacing” the estrogen receptor and stimulating tumor growth [57]. After treatment with EENL we observed a decrease of the *AR* transcripts in the MDA-MB-231 line. This effect, therefore, strongly suggests a possible action of EENL in triple-negative tumors and patients treated with this phenolic compound would have a better prognosis considering the behavior of AR signaling in TNBC [58].

After treatment with EENL–EA and EENL–DCM we detected an increase in *AR-V7* transcripts in MDA-M-231. The *AR-V7* gene is expressed in primary BC and in breast tumor cell lines, which can promote growth and mediate resistance in androgen deprivation therapies (ADT) in BC subsets [59,60,61]. In this context, the expression of *AR* and its variants emerge as a new strategy for BC treatment.

The effects of *AR* are directly linked to the *ESR* pathway [62,63]. ESR+ breast tumor cells, such as MCF7, have the growth stimulated by androgens and inhibited by the antiandrogens [64,65]. AR antagonizes the ESR growth by: (1) directly inhibiting ESR target genes; (2) competing with ESR for binding in the estrogen responsive elements (ERE); (3) sequestering transcriptional factors (TFs); and (4) inducing apoptosis by direct negative regulation of *cyclin D1* gene expression [62,66,67].

Taken together, our results demonstrate a differential effect of EENL in breast tumor cell lines (MCF7 and MDA-MB-231), specially modulating nuclear receptor expression. The behavior of *AR-V7* in the MDA-MB-231 tumor cell line indicates new pathways involved in tumor biology, especially as a therapeutic target. Further studies are needed to better understand the role of these compounds in the modulation of such receptors.

## 4. Materials and Methods

### 4.1. Plant Material

The *Azadirachta indica* (neem) leaves were collected at latitude 18°34′13.99″ S and longitude 46°29′52.57″. The present study was registered in the Genetic Patrimony Management Board (CGEN) (number A2FFD84) and a voucher specimen was recorded in the Herbarium of Institute of Biology of the Federal University of Uberlandia with the number 71869. After identification, the neem leaves were sanitized and oven-dried (Lucaderma), for 72 h at 40 °C. The dry leaves were further milled in a knife mill (Solab).

### 4.2. Ethanolic Extraction and Determination of Phenolic Compounds

Flavonoids and phenolic compounds were extracted according to Cechinel-Filho and Yunes method (1998) [68]. Dried and milled leaves (100 g) were stirred for homogenization in a solution of methanol 50% and protected from light for 20 days.

Extractions were further performed with triple addition of 30 mL of ethyl acetate (EA) P.A. (Proquímicos), and dichloromethane (DCM) P.A. (Alphatec, Carlsbad, CA, USA), separately. After filtration, the filtrates were evaporated in rotary evaporator at 55 °C with reduced pressure and resuspended with DMSO (Sigma Aldrich, St. Louis, MO, USA). All experiments were conducted in six replicates. For in vivo assays, EENL were obtained according to Silva (2010) [69].

Total phenolic compounds (mg gallic acid equivalents (GAE)/g DW) were determined by the Folin–Ciocalteu method [70]. Absorbance analysis was performed in spectrophotometer UV-VIS at the wavelength of 760 nm and dosage was adopted from Swain and Hillis (1959) [71].

### 4.3. In Vitro Assays

#### 4.3.1. Breast Cell Lines

MCF 10A (non-tumorigenic), MCF7 (ESR + BC), and MDA-MB-231 (triple-negative phenotype) breast cells lines were obtained from ATCC and cultured with Dulbecco’s Modified Eagle Medium (DMEM) (Cultilab), Iscove’s Modified Dulbecco’s Medium (IMDM) (Cultilab), and Leibovitz’s L-15 Medium (Sigma Aldrich), respectively. MCF 10A was supplemented with 10% of fetal bovine serum (FBS), 20 ng/mL of epidermal growth factor (EGF), 500 ng/mL of hydrocortisone, 10 µg/mL of insulin, and 50 μg/L of gentamycin. The malignant lineages were supplemented with 10% of FBS and 50 μg/L of gentamycin. All lineages were incubated at 37 °C in a humidified atmosphere containing 5% CO_2_ atmosphere until they reached 80% of confluence.

#### 4.3.2. Antiproliferative Assay

The antiproliferative effect of the Ethanolic Extract from Neem Leaves (EENL) on the three lineages was evaluated by MTT assay (3-(4,5-dimethylthiazol-2-yl)-2,5-diphenyltetrazolium bromide) according to Mosmann (1983) [72]. After confluence, 10^4^ cells per well were seeded onto 96 well plates and incubated for 24 h at 37 °C and 5% CO_2_ atmosphere for adherence.

Treatments were performed in eight concentrations of EENL–EA and EENL–DCM (1 μg/mL; 0.5 μg/mL; 0.25 μg/mL; 0.125 μg/mL; 0.0625 μg/mL; 0.03125 μg/mL; 0.015625 μg/mL; and 0.0078125 μg/mL) for 24 and 48 h. Controls included groups treated with DMSO according to each concentration of the extracts, untreated cells (positive control), and wells with only medium (negative control).

After incubation, 10 µL of MTT (5 mg/mL in PBS) (Sigma Chemical Co., St. Louis, MO, USA) was added in each well and incubated for 4 h. Formazan crystals were dissolved in a Sodium Dodecyl Sulfate (SDS) solution containing *N*-dimethylformamide at 50%. The absorbance was measured at the wavelength of 570 nm in a Thermo Plate Reader (TP-Reader), and the percentage of viable cells was calculated by using the formula in Equation (1).

(1) $$\%\mathrm{Cell}\;\mathrm{Viability}=\frac{\mathrm{SA}-\left(\mathrm{PC}-\mathrm{NC}\right)}{\mathrm{DMSO}-\left(\mathrm{PC}-\mathrm{NC}\right)}\;\times \;100$$

SA = Sample A_570nm_ (treated cells)

PC = Positive Control A_570nm_ (untreated cells)

NC = Negative Control A_570nm_ (culture medium only)

DMSO = DMSO Control A_570nm_

#### 4.3.3. Quantitative Real-Time PCR

Total RNA was extracted before and after treatments with EENL–EA (1.0 μg/mL) and EENL–DCM (0.03125 μg/mL) for 48 h. Trizol Reagent (Thermo Fisher Scientific, Waltham, MA, USA) was used for extraction according to the manufacturer’s recommendations. After isolation, RNA quality was electrophoretic confirmed (1.5% agarose gel) and the concentration was determined using the NanoDrop System (Thermo Fisher Scientific). Reverse transcription was performed as previously described [73].

Quantitative qPCR was performed to evaluate gene expression profiles of *ESR1*, *ESR2*, *AR, AR-V1*, *AR-V4,* and *AR-V7*. For normalization, *Beta-2-microglobulin* (*B2M)* gene was used and Cq method was validated through relative standard curve construction.

Mix solutions containing 5 µL of Power SYBR Green PCR Master Mix (Applied Biosystems, Carlsbad, CA, USA), 5 pmol/µL of primers (Table 2), and 2 µL of cDNA were prepared and gene expression profiles were analyzed in a 7300 Real Time PCR System (Applied Biosystems). Data from MCF 10A was used as calibrator.

### 4.4. In Vivo Assays

*Drosophila melanogaster* is a well-established insect model for human diseases and toxicological research due to its well-documented genetics, developmental biology, and capacity to activate enzymatically promutagens and procarcinogens in vivo [75].

The *Drosophila* tumor suppressor gene warts (wts) encodes a homolog of human myotonic dystrophy kinase and is required for the control of cell shape and proliferation. Homozygous loss of the wts gene of *Drosophila*, caused by mitotic recombination in somatic cells, leads to the formation of tumors, with aspects of warts appearing on the body of adult flies [76].

#### 4.4.1. Chemical Agents

Doxorubicin (DXR; CAS 25316-40-9) was used as positive control, commercially known as ADRIBLASTINA^®^, manufactured and packaged by Actavis Italy S.p.A–Nerviano, Milan–Italy; registered, imported and distributed by Laboratories Pfizer LTDA, Pharmacist responsible—José Cláudio Bumerad—CRF-SP n° 43746, CNPJ n° 46070 868/0001-69. Ethanol 2.38% was used as the negative control and to dilute the EENL. DXR, in the concentration used in this work, previously generated reactive oxygen species, induced homologous recombination in *D. melanogaster,* and tumor formation [77,78,79,80,81].

#### 4.4.2. Test for the Detection of Epithelial Tumor Clones (Warts)

The test warts (*wts*) was performed using two strains of *Drosophila melanogaster* (*Diptera: Drosophilidae*): (1) Warts lineage (*wts/TM3*, *Sb^1^*) presents a lethal allele *wts*, on chromosome three, balanced by a chromosome TM3, which is characterized by multiple inversions and marked by dominant mutated stubble (Sb). The stubble mutation is phenotypically characterized by short and thicker hair identified all over the body of the fly. This lineage was kindly provided by the Bloomington *Drosophila* Stock Center, from Indiana University-USA, registered: Bloomington/7052; (2) Lineage flies multiple wing hairs (mwh) has the marker gene on the chromosome 3 (3-0.3) in a distal position, which is characterized by expressing three or more hairs in each cell. This lineage was kindly provided by Dr. Ulrich Graf (Physiology and Animal Husbandry, Institute of Animal Science, ETH Zurich, Schwerzenbach, Switzerland) [75].

Stocks are grown at the Laboratory of Cytogenetics and Mutagenesis (University Center of Patos de Minas-UNIPAM, Brazil), kept in jars with culture medium for *D. melanogaster*. The medium is composed of 820 mL of water, 25 g of yeast powder (*Sacchoromyces cerevisae*), 11 g of agar, 156 g of banana, and 1 g of nipagin. The lineages are maintained inside an incubator B.O.D. 411 D, under light/dark cycles (12 h/12 h), at 25 ± 1 °C and approximately 60% humidity.

#### 4.4.3. Experimental Procedure

To obtain *wts +/+ mwh* heterozygotic larvae, virgin females *wts/TM3*, *Sb^1^* [82] are crossed with *mwh/mwh* males [83]. The eggs of the descendants were collected for 8 h in flasks containing a culture medium suitable for posture (4% *w*/*v* agar-agar based, topped with a thick layer of live baker’s yeast supplemented with sucrose). After 72 ± 4 h, third-instar larvae were washed in reverse osmosis water and collected using a fine mesh sieve. The larvae were transferred to a 25 mL vial containing 1.5 g of instant mashed potatoes (HIARI^®^ brand, São Paulo, Brazil) culture medium. Five milliliters of EENL (in the concentrations 0.03125%, 0.0625%, and 0.125%), diluted in 2.38% ethanol, were added to each tube on its own as well as together with DXR. A co-treatment system was used to apply DXR and EENL. Ethanol 2.38% was used as negative control, and DXR 0.4 mM as the positive control (Table 3). The treatments were performed in quadruplicate.

These larvae were submitted to a chronic treatment for 48 h. After 120 h of the larval feeding, following metamorphosis, the flies were collected and preserved in 70% ethanol.

#### 4.4.4. Analysis of the Flies

Adult flies of the *wts +/+ mhw* genotype with long hairs were analyzed for the presence of tumor (wart). The adults with short and thick hairs were discarded, since they did not have the gene being studied. The flies were observed using a stereoscopic magnifying glass and entomological tweezers. Only tumors large enough to be unequivocally classified were recorded. The tumor frequency was calculated as the number of tumors divided by number of *wts +/+ mwh* flies [75]. A total of 1600 flies were analyzed, 200 flies for each control (positive or negative), 200 flies for each isolated concentration, and 200 flies for each associated group.

### 4.5. Statistical Analysis

Statistical analysis was carried out using GraphPad Prism 7.0 (GraphPad Software, La Jolla, CA, USA). *P* < 0.05 was considered significant. Paired Student *t* test was used to compare gene expression and the unpaired for phenols dosage. MTT assays were tested using ANOVA. Finally, the carcinogenic potential of EENL in the *D. melanogaster* model was validated by the Mann–Whitney and Wilcoxon nonparametric U test, using α = 0.05 level of significance.

## Figures and Tables

**Table 1:** Frequency of tumors in the different segments of *Drosophila melanogaster.* The flies used in this study are heterozygous for the warts tumor suppressor gene and were treated with different concentrations of isolated Ethanolic Extract from Neem Leaves (EENL) and EENL associated with doxorubicin (DXR).

Treatments	Indiv. (N)	Tumors						
		Eyes	Head	Wings	Body	Legs	Halteres	Total Tumors
Negative Control	203	0.00 (00)	0.00 (01)	0.00 (00)	0.00 (01)	0.01 (02)	0.00 (01)	0.02 (05)
Positive Control	176	0.01 (02)	0.04 (07)	0.20 (36)	0.15 (26)	0.05 (09)	0.01 (01)	0.46 (81) +
EENL 0.03125%	200	0.00 (00)	0.01 (02)	0.10 (20)	0.04 (08)	0.03 (06)	0.03 (05)	0.21 (41) +
EENL 0.0625%	200	0.02 (03)	0.01 (02)	0.07 (13)	0.03 (06)	0.04 (07)	0.01 (01)	0.16 (32) +
EENL 0.125%	218	0.00 (00)	0.01 (02)	0.04 (08)	0.13 (29)	0.01 (03)	0.01 (05)	0.20 (44) +
EENL 0.03125% + DXR	200	0.01 (01)	0.26 (52)	2.08 (416)	0.98 (196)	1.92 (384)	0.19 (37)	5.43 (1086) **
EENL 0.0624% + DXR	200	0.09 (18)	0.59 (117)	3.65 (730)	1.82 (363)	3.65 (729)	0.45 (90)	10.24 (2047) **
EENL 0.125% + DXR	165	0.03 (05)	0.06 (10)	0.24 (39)	0.28 (46)	0.06 (10)	0.02 (04)	0.69 (114) **

Statistical diagnosis according to the Mann–Whitney *U*-Test. Level of significance *P* ≤ 0.05. + value considered statistically different from the negative control (*P* ≤ 0.05). ** value considered statistically different from the positive control (DXR 0.4 mM) (*P* ≤ 0.05). DXR = doxorubicin (0.4 mM). EENL = Ethanolic Extract from Neem Leaf.

**Table 2:** Oligonucleotide sequences used for qPCR assays.

GENE	Primers sequence (Forward/Reverse) (5′–3′)
*β2M* [73]	F: CCTGCCGTGTGAACCATGT/R: GCGGCATCTTCAAACCTCC
*ESR1*	F: CTAACTTGCTCTTGGACAGGAAC/R: GATTTGAGGCACACAAACTCCTC
*ESR2*	F: GGGAATGGTGAAGTGTGGCT/R: TCATGTGTACCAACTCCTTGTCGG
*AR*	F: CATGTGGAAGCTGCAAGGTCT/R: GTGTAAGTTGCGGAAGCCAGG
*AR-V1* [74]	F: CTACTCCGGACCTTACGGGGACATGCG/R: GATTCTTTCAGAAACAACAACAGCTGCT
*AR-V4* [74]	F: CTACTCCGGACCTTACGGGGACATGCG/R: CTTTTAATTTGTTCATTCTGAAAAATCCTC
*AR-V7* [74]	F: CTACTCCGGACCTTACGGGGACATGCG/R: TGCCAACCCGGAATTTTTCTCCC

**Table 3:** *Drosophila melanogaster* larvae treated with different concentrations of EENL. EENL were used isolated and associated with doxorubicin.

Treatments	Concentrations	Composition
**Negative control (−)**	-	MP + 5 mL ROW + 2.38% of ethanol P.A
**Positive control (+)**	-	MP + DXR 0.4 mM
**T1**	0.03%	MP + 5 mL of EENL 0.03125%
**T2**	0.06%	MP + 5 mL of EENL 0.0625%
**T3**	0.13%	MP + 5 mL of EENL 0.125%
**T4**	0.03125% + DXR	MP + 5 mL of EENL 0.03125% + DXR 0.4 mM
**T5**	0.0625% + DXR	MP + 5 mL of EENL 0.0625% + DXR 0.4 mM
**T6**	0.125% + DXR	MP + 5 mL of EENL 0.125% + DXR 0.4 mM

MP = Mashed Potatoes. ROW = Reverse Osmose Water. T1-6 = Treatments. EENL = Ethanolic Extract from Neem Leaves. DXR = Doxorubicin. P.A = Pure for Analysis.

## Abbreviations

BC	Breast cancer
neem	*Azadirachta indica*
DCM	Dichloromethane
EA	Ethyl acetate
EENL	Ethanolic extracts of neem leaves
DXR	Doxorubicin
ESR1	Estrogen receptor alpha
ESR2	Estrogen receptor beta
AR	Androgen receptor
wts	Test warts
